# Did hardening occur among smokers in England from 2000 to 2010?

**DOI:** 10.1111/add.12359

**Published:** 2013-10-28

**Authors:** Graeme Docherty, Ann McNeill, Coral Gartner, Lisa Szatkowski

**Affiliations:** 1 Division of Epidemiology and Public Health, UK Centre for Tobacco Control Studies, University of NottinghamNottingham, UK; 2 UK Centre for Tobacco Control Studies, National Addiction Centre, Institute of Psychiatry, King's College LondonLondon, UK; 3 Centre for Clinical Research, University of Queensland, Royal Brisbane and Women's Hospital SiteHerston, Qld, Australia

**Keywords:** Cessation, dependence, hardcore, hardening, inequalities, population

## Abstract

**Aims** To assess trends in the prevalence of ‘hardcore’ smoking in England between 2000 and 2010, and to examine associations between hardcore smoking and socio-demographic variables.

**Design** Secondary analysis of data from the United Kingdom's General Lifestyle Survey (GLF) and the Health Survey for England (HSE).

**Setting** Households in England.

**Participants** Self-reported adult current smokers resident in England aged 26 years and over.

**Measurements** Hardcore smokers were defined in three ways: smokers who do not want to quit (D1), those who ‘usually’ smoke their first cigarette of the day within 30 minutes of waking (D2) and a combination of D1 and D2, termed D3. Multivariable logistic regression was used to explore associations between these variables and calendar year, age, sex and socio-economic status, and *P*-values for trends in odds were calculated.

**Findings** The odds of smokers being defined as hardcore according to D3 increased over time in both the GLF (*P* < 0.001) and HSE (*P* = 0.04), even after adjusting for risk factors. Higher dependence (D2) was noted in men [odds ratio (OR): 1.19, 95% confidence interval (CI): 1.13–1.24], those of 50–59 years (OR: 1.94, 95% CI: 1.80–2.09) and smokers in lower occupational groups (OR: 2.11, 95% CI: (1.97–2.26). Lack of motivation to quit (D1) increased with age and was more likely in men.

**Conclusions** The proportion of smokers in England with both low motivation to quit and high dependence appears to have increased between 2000 and 2010, independently of risk factors, suggesting that ‘hardening’ may be occurring in this smoker population.

## Introduction

Smoking prevalence in the United Kingdom and most industrialized countries has decreased considerably since the 1970s as a result of increasing awareness of the health effects of tobacco and the introduction of tobacco control measures [1]. However, during the past three decades, this fall in prevalence has slowed noticeably in the United Kingdom [1]. Some commentators have suggested that the slower decline in prevalence is due to those smokers, probably less dependent, who find it easiest to quit having done so, thus leaving a group that are more resistant to quitting continuing to smoke. This theory is known as the ‘hardening’ hypothesis, and its premise has attracted attention [2,3]. Although the theory does not take smoking uptake into account, the hardening hypothesis has been tested in the smoking populations of North America, Australia and western Europe, with most studies having limitations including restrictions in the available data [4–6].

By examining hardening in the context of cessation rates in trials [7], Irvin & Brandon found a decrease in quit rates in smokers registered in cessation trials in the United States between 1975 and 1998, which may be regarded as evidence for hardening. However, these data relate to motivated smokers already making a registered quit attempt and, by definition, excludes those who do not want or intend to make a quit attempt. Examining cessation rates alone limits one's approach to the subject [8].

In contrast, Fagerström & Furberg [9] used the Fagerström Test for Nicotine Dependence (FTND) to examine dependence trends in several developed countries. By using a variety of retrospective data sources, they found that smoking prevalence was generally correlated inversely with FTND score. As prevalence decreased, the overall level of dependence in continuing smokers increased, suggesting that hardening may be occurring in these countries. However, some have pointed out that their findings should be interpreted with caution due to the use of often heterogeneous and occasionally non-representative data sets in their analysis [10].

Other studies have utilized the concept of the ‘hardcore smoker’ as a marker of whether hardening is occurring in the population, but there is no universally agreed definition of what constitutes a hardcore smoker [11]. Table 1 shows the definitions of hardcore used in previous research, along with data sources and associated prevalence [4–6,12–14]. Not all studies define hardcore *per se*, but assess hardening in terms of other factors such as poor mental health, which are likely to be associated with hardcore smoking [15,16].

**Table 1:** Construct definitions of hardcore smoking

*Authors (year of publication)*	*Location*	*Data source and year(s)*	*Definition of hardcore*	*Notable exclusions*	*Hardcore prevalence as % of all smokers*
Sorg *et al*. (2011) [6]	USA: Missouri	Missouri County level study, representative survey, 2007	No quit attempt in last 12 months	Smokers 25 years or under	7.8%
			CPD 15 or more		
			Do not intend to quit in future		
Lund *et al*. (2011) [5]	Norway	Statistics Norway: national cross-sectional survey, 1996–2009	No quit attempt in previous 12 months	Smokers 25 years or under	11% average 1996–2009 (max 16%, min 6%)
			No intention to quit in next 6 months		
			Belief in continued smoking in 5 year's time		
MacIntosh & Coleman (2006) [12]	UK	Secondary research data. General practice, Leicestershire, UK, 1995–96, 1998–99	Do not intend to quit in next month		16.1% (14.1–18.4)
			No desire to quit		
			No quit attempts longer than 24 hours in past year		
Jarvis *et al*. (2003) [13]	UK	UK Health Education Authority surveys, 1994–97	Do not intend to quit in future	Smokers 25 years or under	15.7%
			No desire to quit		
			No quit attempts in past year		
			less than 1 day without smoking cigarettes over past 5 years		
Augustson & Marcus (2004) [4]	USA: national	1998–99 Tobacco Use Supplement to Current Population Survey (TUS-CPS)	Do not intend to quit in next 6 months	Smokers 25 years or under	13.7% of current smokers
			No previous quit attempt		
			CPD 15 or more		
			Daily smoker, with minimum 5 years smoking history		
Emery *et al*. (2000) [14]	USA: California	California Tobacco Survey, 1996	Expect to never quit	Smokers 25 years or under	5.2%
			No quit attempts in past year		
			CPD 15 or more		

CPD: cigarettes per day.

Most studies have conflated dependence and motivation in their definitions of hardcore or hardening. Our aim was to separate motivation and dependence factors by creating variables that examine each construct separately and together in one ‘hardcore’ definition, using general population data sets that have not been examined previously in this context. Our research questions were (i) to assess the prevalence and trends between 2000 and 2010 of three categories of smokers that could be described as hardcore smokers and (ii) to examine associations between hardcore and socio-demographic variables.

## Methods

### Data

Our data were two cross-sectional surveys: the English subset of the UK General Lifestyle Survey (GLF) and the Health Survey for England (HSE), using data from 2000 to 2010 [17,18]. Both surveys use computer-assisted personal interviewing to question individuals aged 16+ years living in private households, asking questions on a range of topics including smoking behaviour and tobacco consumption. Annual response rates in 2000–10 ranged from 68 to 72% in the GLF, and 58 to 68% in the HSE. The anonymized data sets were obtained from the UK Data Archive (http://ukdataservice.ac.uk) and no additional ethical approval was required for their use. Both survey data sets were analysed separately as each are of sufficient size to estimate effects with precision, and this allowed us to replicate our findings in two independent data series.

### Inclusion criteria and hardcore variables

Only current smokers 26 years of age and over were included in the analysis, as evidence suggests that younger smokers are less likely to be habitual in their smoking behaviour [19], and this age corresponds to the lower limit in several other studies of hardcore smoking [4–6]. From the smoking variables available on the data sets, we used three variable definitions of what could be described as hardcore smoking. Low motivation (termed D1) included current smokers who reported ‘no’ to the question: ‘would you like to give up smoking altogether?’. Highly dependent smokers (D2) were identified as those current smokers who answered ‘30 minutes or less’ to the question: ‘How soon after waking do you usually smoke your first cigarette?’. D3 included those who answered affirmatively to D1 and D2. Smokers with missing data for either or both of D1 or D2 were excluded from the analysis of D3. D1 and D2 were studied independently as they represent low motivation to quit and high dependence, respectively, each needing to be considered in assessments of hardening.

We used time to first cigarette (TTFC) as the preferred measure of dependence, rather than cigarettes per day (CPD) or any combination of these two measures such as the Heaviness of Smoking Index [20], due to CPD being increasingly less reliable as a measure of dependence because of the effects of smoke-free laws and taxation-related price rises [21,22]. These policies are known to decrease consumption without necessarily reducing dependence, and evidence suggests that TTFC is an important predictor of quitting [23].

### Statistical analysis

For each year of the GLF and HSE we calculated the percentage of smokers identified as meeting each of the three definitions and presented these graphically. Additionally, we fitted univariable and multivariable logistic regression models to investigate the demographic and socio-economic factors associated with being in these groups and tested for linear trends in odds across our ordered categorical explanatory variables (age group, socio-economic status and year). In these models, socio-economic status was defined by the standard occupational classification for the United Kingdom, with ‘high’ status comprising professional and managerial occupations; ‘intermediate’ comprising skilled non-manual and skilled manual workers; and ‘low’ encompassing semi-skilled and unskilled occupations [24]. Non-responders and any other non-income-generating categories (e.g. unemployed, retired) were coded as missing. Similar findings were observed for two surveys so, for brevity, only findings from the GLF are presented here, with results from the HSE available as Supporting information online; any major differences between the two surveys are mentioned in the text.

Analyses were undertaken in Stata version 11.0 (StataCorp LP, College Station, TX, USA).

## Results

**Figure 1:**
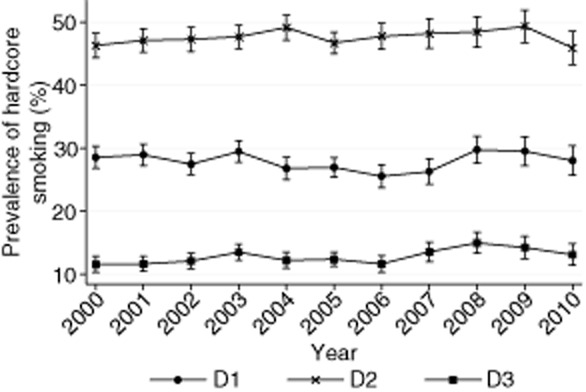
D1–D3 prevalence in the General Lifestyle Survey 2000–10

According to GLF data, adult smoking prevalence in England declined from 27% in 2000 to 20% in 2010 [17]. The proportion of smokers each year who satisfied each of our definitions of hardcore in the GLF are shown in Fig. 1, with the average prevalence over the study period being 27.9% for D1, 47.6% for D2 and 12.8% for D3. Only D1 in the GLF had an appreciable number of missing cases, the proportion increasing over time from 3.9% in 2000 to 7.8% in 2010 (*P* < 0.001).

Tables 2–4 show the associations between each of the defined hardcore variables and socio-demographic characteristics, including year. D3 smokers showed an increasing trend between 2000 and 2010, following adjustment (GLF, *P* < 0.001; HSE, *P* = 0.04) with no significant trend found for D1 and D2. For D3, the difference between unadjusted and adjusted odds across years was minimal.

**Table 2:** Predictors of D1 smoker in General Lifestyle Survey 2000–10

	*Odds of fulfilling definition D1 criteria 2000–10*				
	*n*	*Unadjusted OR (95% CI)*	*P-value for trend*	*Adjusted OR^a^ (95% CI)*	*P-value for trend*
Gender					
Female	13 530	1.00	0.155	1.00	0.026
Male	12 755	1.04 (0.99–1.10)		1.06 (1.01–1.12)	
Age group (years)					
26–34	5 866	1.00	<0.001	1.00	<0.001
35–49	9 796	1.07 (0.99–1.15)		1.07 (0.99–1.15)	
50–59	5 116	1.40 (1.29–1.53)		1.41 (1.29–1.53)	
60+	5 507	2.60 (2.40–2.82)		2.59 (2.39–2.81)	
Socio-economic status					
High	6 565	1.00	<0.001	1.00	0.001
Intermediate	11 817	1.05 (0.98–1.12)		1.00 (0.93–1.07)	
Low	6 800	1.18 (1.10–1.27)		1.08 (1.00–1.17)	
Missing^b^	1 103	1.19 (1.04–1.37)		1.26 (1.10–1.45)	
Survey year					
2000	2 537	1.00	0.104	1.00	0.760
2001	2 703	1.04 (0.93–1.17)		1.04 (0.92–1.17)	
2002	2 525	0.96 (0.85–1.09)		0.96 (0.85–1.09)	
2003	2 974	1.04 (0.92–1.16)		1.04 (0.93–1.17)	
2004	2 430	0.92 (0.81–1.04)		0.92 (0.82–1.05)	
2005	3 360	0.92 (0.82–1.03)		0.91 (0.81–1.02)	
2006	2 356	0.88 (0.78–1.00)		0.87 (0.77–0.98)	
2007	2 023	0.96 (0.84–1.09)		0.93 (0.81–1.06)	
2008	1 953	1.12 (0.98–1.27)		1.06 (0.93–1.21)	
2009	1 775	1.13 (1.00–1.29)		1.04 (0.91–1.19)	
2010	1 649	1.10 (0.96–1.26)		1.01 (0.88–1.16)	

a Adjusted for all other variables in the table.

b Missing data excluded from test for trend in odds. OR: odds ratio; CI: confidence interval.

**Table 3:** Predictors of D2 smoker in General Lifestyle Survey 2000–10

	*Odds of fulfilling definition D2 criteria 2000–10*				
	*n*	*Unadjusted OR (95% CI)*	*P-value for trend*	*Adjusted OR^a^ (95% CI)*	*P-value for trend*
Gender					
Female	14 324	1.00	<0.001	1.00	<0.001
Male	13 468	1.11 (1.05–1.16)		1.19 (1.13–1.24)	
Age group (years)					
26–34	6 166	1.00	<0.001	1.00	<0.001
35–49	10 290	1.63 (1.53–1.74)		1.65 (1.54–1.76)	
50–59	5 427	1.95 (1.81–2.10)		1.94 (1.80–2.09)	
60+	5 909	1.38 (1.29–1.49)		1.32 (1.22–1.42)	
Socio-economic status					
High	6 881	1.00	<0.001	1.00	<0.001
Intermediate	12 497	1.54 (1.45–1.63)		1.56 (1.47–1.66)	
Low	7 247	2.02 (1.89–2.16)		2.11 (1.97–2.26)	
Missing^b^	1 167	1.72 (1.52–1.95)		1.86 (1.64–2.11)	
Survey year					
2000	2 632	1.00	0.091^b^	1.00	0.288
2001	2 868	1.05 (0.95–1.17)		1.04 (0.93–1.16)	
2002	2 656	1.06 (0.95–1.19)		1.06 (0.95–1.18)	
2003	3 114	1.09 (0.98–1.21)		1.08 (0.97–1.20)	
2004	2 564	1.14 (1.02–1.27)		1.12 (1.00–1.25)	
2005	3 545	1.03 (0.93–1.14)		1.01 (0.91–1.12)	
2006	2 491	1.08 (0.96–1.20)		1.05 (0.94–1.17)	
2007	2 175	1.10 (0.98–1.24)		1.08 (0.96–1.21)	
2008	2 072	1.12 (1.00–1.26)		1.10 (0.97–1.23)	
2009	1 902	1.15 (1.02–1.29)		1.12 (0.99–1.26)	
2010	1 773	1.05 (0.93–1.18)		1.02 (0.90–1.16)	

a Adjusted for all other variables in the table.

b Missing data excluded from test for trend in odds. OR: odds ratio; CI: confidence interval.

**Table 4:** Predictors of D3 smoker in General Lifestyle Survey 2000–10

	*Odds of fulfilling definition D3 criteria 2000–10*					
	*n*	*Unadjusted OR (95% CI)*	*P-value for trend*	*Adjusted OR^a^ (95% CI)*	*P-value for trend*
Gender					
Female	13 501	1.00	0.002	1.00	<0.001
Male	12 719	1.12 (1.04–1.20)		1.20 (1.12–1.29)	
Age group (years)					
26–34	5 853	1.00	<0.001	1.00	<0.001
35–49	9 773	1.39 (1.25–1.55)		1.38 (1.24–1.54)	
50–59	5 102	1.94 (1.73–2.19)		1.92 (1.71–2.16)	
60+	5 492	2.48 (2.21–2.77)		2.36 (2.11–2.64)	
Socio-economic status					
High	6 545	1.00	<0.001	1.00	<0.001
Intermediate	11 790	1.41 (1.28–1.56)		1.38 (1.25–1.52)	
Low	6 783	1.91 (1.73–2.12)		1.86 (1.68–2.06)	
Missing^b^	1 102	1.79 (1.50–2.14)		1.91 (1.59–2.29)	
Survey year					
2000	2 531	1.00	<0.001	1.00	<0.001
2001	2 696	1.05 (0.89–1.24)		1.04 (0.88–1.23)	
2002	2 520	1.09 (0.92–1.29)		1.09 (0.92–1.29)	
2003	2 965	1.22 (1.04–1.42)		1.21 (1.03–1.42)	
2004	2 425	1.10 (0.93–1.30)		1.10 (0.93–1.30)	
2005	3 356	1.10 (0.94–1.29)		1.09 (0.93–1.28)	
2006	2 354	1.05 (0.88–1.25)		1.03 (0.86–1.22)	
2007	2 020	1.30 (1.09–1.54)		1.26 (1.06–1.50)	
2008	1 945	1.43 (1.20–1.69)		1.36 (1.15–1.62)	
2009	1 771	1.40 (1.18–1.67)		1.30 (1.09–1.55)	
2010	1 637	1.30 (1.09–1.56)		1.21 (1.01–1.45)	

a Adjusted for all other variables in the table.

b Missing data excluded from test for trend in odds. OR: odds ratio; CI: confidence interval.

Men were more likely than women to satisfy each criterion in the adjusted analyses. Increasing age was a risk factor for each definition with, for example, those aged 60+ being more than twice as likely to meet the definition of D3 compared to those aged 26–34 [adjusted odds ratio (OR): 2.36, 95% confidence interval (CI): 2.11–2.64]. The odds of smokers meeting the criteria for all three definitions increased with decreasing socio-economic status, although the trend was only just statistically significant in the case of D1.

The HSE produced similar findings (see Supporting information), with the exception of there being no association between socio-economic status and meeting the criterion for D1.

## Discussion

### Summary of results

There is an apparent increase in the proportion of smokers satisfying D3 criteria combining low motivation and high dependence over the study period, although when low motivation (D1) and high dependence (D2) were assessed separately no trends were noted in either of D1 or D2 across the 11-year period. The prevalence of D3 approximated 10–15%, with those not motivated to quit (D1, ∼30%) consistently lower than the proportion classed as highly dependent (D2, ∼50%). Males and older age groups and those with lower incomes were more likely not to want to quit and have higher tobacco dependence.

### Limitations

The surveys we utilized do not have data on other relevant smoking attributes such as previous quit attempts or how long a person has smoked. However, as most smokers start in adolescence, restricting our analysis to smokers aged 26 years and over would ensure that most had been smoking for at least 5 years. We used not *wanting* to quit as our motivation variable, but recognize that this may not always yield the same response as the question: ‘do you *intend* to quit?’ which was not asked in either survey. However, a previous study found that intention and desire to quit both predicted quit attempts, so we believe that using desire is appropriate here [25]. Participants' responses to questions on smoking behaviour in each survey are based on self-report, and evidence suggests that such methods are likely to underestimate true smoking prevalence in populations [26]. This is because people may be reluctant to report cigarette smoking due to increased undesirability or stigma associated with smoking. Response rates in the surveys were somewhat low, ranging from 59 to 75%, and this may have introduced some non-response bias, although weighting for non-responders has been taken into account.

### Strengths

The two surveys are regarded as sources of official data on household and health-related behaviour [17,18], and are representative of the population in England. As repeat cross-sectional surveys of adequate size, they provide an appropriate tool for examining hardening in the general population of smokers over time.

### Wider discussion of literature

Our findings for D1 and D2 separately corroborate other studies that have not observed an upward trend in the proportion of hardcore smokers [5,15]. However, the results for the combined D3 variable suggest that there may well be an increasing prevalence of hardcore smokers, who are both highly dependent and unmotivated to quit, in the English smoking population between 2000 and 2008 with a suggestion of a downturn since that time. As can be seen by the adjusted regression analysis and a comparison of the unadjusted and adjusted estimates of D3 trends across survey years, including demographic and socio-economic variables as covariates in the analysis had only a minimal effect on the unadjusted estimates, suggesting that the increase in D3 prevalence over the study period is not due primarily to an increase of those in ‘high risk’ groups who are more likely to be heavily addicted or have lower motivation to quit, e.g. older smokers or those on lower incomes. The increasing trend is more likely to reflect a true increase in the proportion of smokers satisfying our D3 criteria, dispersed across socio-demographic groups.

During the study period a number of tobacco control policies were introduced in England, including prohibitions on print and cinema advertising and sports sponsorship, and bans of smoking in public places. While the impact (if any) of these policies on hardcore smoking is not able to be ascertained from these results, it is interesting to note that the main increase occurred between 2006 and 2008, coinciding with the implementation of smoke-free legislation in 2007, which may have played a role in increasing resistance among highly dependent smokers, also evidenced by an upward trend in not wanting to stop. The subsequent downward trend in D3 corresponded to the start of a lengthy financial recession that began in late 2008 [27]. Worsening economic conditions may be associated with a drop in heavy dependent smoking (D2), perhaps by reducing consumption. Previous research on smoking behaviour during economic recessions has been inconclusive as to whether or not smoking increases or decreases [28–30]. While there is evidence that desire to quit is lower among low-income groups [31], these studies do not examine the impact of a change in income on desire to quit. Further research is needed to explore the reasons for the changing trends in hardcore smoking and, in particular, whether the upturn reported here is sustained.

The association of high dependence with lower occupational groups is consistent with other research [32,33], providing further evidence of the socio-economic inequalities in smoking [34], although other markers of inequalities, such as mental health, are not reported here.

### Practical implications

Our findings suggest that motivation to quit in the smoker population has not been greatly affected by tobacco control measures, which tobacco control advocates need to be aware of when considering measures to encourage quit attempts; nor has dependence, as measured by TTFC, increased markedly. With the proportion highly dependent being much greater than those with low motivation, measures targeted specifically at high dependence groups may therefore be warranted with the aim of reducing dependency in the smoker population. A similar recommendation was made in a recent European study [35], that found that female smokers in countries with well-developed tobacco control policies, such as Ireland and Sweden, tended to have higher dependence than those in countries yet to establish these. Targeted measures could include harm reduction interventions such as the provision of pharmacotherapies for reduction as a first step towards quitting, or more targeted and tailored mass media campaigns for heavy smokers.

Future research should continue to assess separately motivation and dependence factors as well as these factors in combination, in order to improve our understanding of trends in the variables contributing to hardcore smoking. Improvement of variables used to define each of these factors could be considered, for instance by recommending standard questions to be included in surveys. This would enable comparisons across surveys and regions, and thus refine our understanding.

## Supporting information

**Figure S1** D1–D3 prevalence in the Health Survey for England 2000–10 **Table S1** Adjusted and unadjusted odds ratios, including *P*-values of predictors of D1 smokers in the Health Survey for England 2000–10. **Table S2** Adjusted and unadjusted odds ratios, including *P*-values of predictors of D2 smokers in the Health Survey for England 2000–10. **Table S3** Adjusted and unadjusted odds ratios, including *P*-values of predictors of D3 smokers in the Health Survey for England 2000–10.
