# Supplementary material for: Did hardening occur among smokers in England from 2000 to 2010?
Source: Addiction. 2013 Oct 28;109(1):147–54. doi: 10.1111/add.12359 (PMC3933730; doi:10.1111/add.12359)
Supplement: Supplementary file 1 — Figure S1 D1–D3 prevalence in the Health Survey for England 2000–10 Table S1 Adjusted and unadjusted odds ratios, including P-values of predictors of D1 smokers in the Health Survey for England 2000–10. Table S2 Adjusted and unadjusted odds ratios, including P-values of predictors of D2 smokers in the Health Survey for England 2000–10. Table S3 Adjusted and unadjusted odds ratios, including P-values of predictors of D3 smokers in the Health Survey for England 2000–10. [file add0109-0147-sd1.doc]

**Figure 1: D1-D3 prevalence in the Health Survey for England 2000-2010**

| **Table 1: Adjusted and unadjusted Odds Ratios, including p values of predictors of D1 smokers in the Health Survey for England 2000-2010** | | | | | |
| --- | --- | --- | --- | --- | --- |
|  | Odds of fulfilling definition D1 criteria 2000-2010 | | | | |
|  | n | Unadjusted OR  (95% CI) | p-value for trend | Adjusted OR^a^  (95% CI) | p-value for trend |
| **Gender** | | | | | |
| Female | 11332 | 1.00 | 0.091 | 1.00 | 0.011 |
| Male | 9924 | 1.05 (0.99-1.11) |  | 1.08 (1.02-1.15) |  |
| **Age group** | | | | | |
| 26-34 | 4793 | 1.00 | <0.001 | 1.00 | <0.001 |
| 35-49 | 8081 | 1.11 (1.02-1.20) |  | 1.11 (1.02-1.21) |  |
| 50-59 | 4064 | 1.47 (1.34-1.62) |  | 1.49 (1.35-1.63) |  |
| 60+ | 4318 | 2.73 (2.50-2.98) |  | 2.75 (2.51-3.01) |  |
| **Socioeconomic status** | | | | | |
| High | 5213 | 1.00 | 0.004 | 1.00 | 0.179 |
| Intermediate | 8415 | 1.00 (0.93-1.08) |  | 0.93 (0.86-1.00) |  |
| Low | 5667 | 1.10 (1.02-1.20) |  | 1.01 (0.93-1.10) |  |
| Missing^b^ | 1961 | 1.11 (0.99-1.24) |  | 1.22 (1.01-1.46) |  |
| **Survey year** | | | | | |
| 2000 | 1764 | 1.00 | 0.042 | 1.00 | 0.592 |
| 2001 | 3290 | 1.02 (0.90-1.15) |  | 1.01 (0.89-1.15) |  |
| 2002 | 1580 | 1.13 (0.98-1.31) |  | 1.14 (0.98-1.32) |  |
| 2003 | 3061 | 0.99 (0.87-1.12) |  | 0.98 (0.86-1.12) |  |
| 2004 | 1302 | 0.96 (0.83-1.13) |  | 0.95 (0.81-1.11) |  |
| 2005 | 1546 | 0.91 (0.78-1.06) |  | 0.90 (0.78-1.05) |  |
| 2006 | 2622 | 1.04 (0.92-1.19) |  | 1.01 (0.89-1.16) |  |
| 2007 | 1267 | 0.99 (0.84-1.15) |  | 0.95 (0.81-1.11) |  |
| 2008 | 2650 | 1.16 (1.02-1.32) |  | 1.13 (0.99-1.29) |  |
| 2009 | 818 | 1.18 (0.99-1.41) |  | 1.13 (0.95-1.35) |  |
| 2010 | 1356 | 1.07 (0.92-1.24) |  | 0.82 (0.65-1.03) |  |
| ^a^ adjusted for all other variables in the table  ^b^ missing data excluded from test for trend in odds | | | | | |

| **Table 2: Adjusted and unadjusted Odds Ratios, including p values of predictors of D2 smokers in the Health Survey for England 2000-2010** | | | | | |
| --- | --- | --- | --- | --- | --- |
|  | Odds of fulfilling definition D2 criteria 2000-2010 | | | | |
|  | n | Unadjusted OR  (95% CI) | p-value for trend | Adjusted OR^a^  (95% CI) | p-value for trend |
| **Gender** | | | | | |
| Female | 11332 | 1.00 | <0.001 | 1.00 | <0.001 |
| Male | 9924 | 1.12 (1.06-1.18) |  | 1.19 (1.12-1.26) |  |
| **Age group** | | | | | |
| 26-34 | 4793 | 1.00 | <0.001 | 1.00 | <0.001 |
| 35-49 | 8081 | 1.58 (1.47-1.70) |  | 1.61 (1.50-1.73) |  |
| 50-59 | 4064 | 1.92 (1.76-2.09) |  | 1.91 (1.76-2.09) |  |
| 60+ | 4318 | 1.34 (1.24-1.46) |  | 1.28 (1.18-1.40) |  |
| **Socioeconomic status** | | | | | |
| High | 5213 | 1.00 | <0.001 | 1.00 | <0.001 |
| Intermediate | 8415 | 1.55 (1.45-1.66) |  | 1.56 (1.46-1.68) |  |
| Low | 5667 | 2.17 (2.01-2.34) |  | 2.22 (2.05-2.40) |  |
| Missing^b^ | 1961 | 1.69 (1.52-1.88) |  | 2.47 (2.08-2.94) |  |
| **Survey year** | | | | | |
| 2000 | 1764 | 1.00 | 0.069 | 1.00 | 0.785 |
| 2001 | 3290 | 1.07 (0.95-1.20) |  | 1.07 (0.95-1.20) |  |
| 2002 | 1580 | 1.14 (1.00-1.31) |  | 1.15 (1.00-1.32) |  |
| 2003 | 3061 | 1.23 (1.09-1.38) |  | 1.23 (1.09-1.38) |  |
| 2004 | 1302 | 1.17 (1.01-1.35) |  | 1.18 (1.02-1.36) |  |
| 2005 | 1546 | 1.21 (1.05-1.39) |  | 1.21 (1.05-1.39) |  |
| 2006 | 2622 | 1.19 (1.05-1.34) |  | 1.16 (1.02-1.31) |  |
| 2007 | 1267 | 1.03 (0.89-1.20) |  | 1.00 (0.86-1.16) |  |
| 2008 | 2650 | 1.14 (1.01-1.29) |  | 1.13 (1.00-1.28) |  |
| 2009 | 818 | 1.30 (1.10-1.54) |  | 1.29 (1.09-1.52) |  |
| 2010 | 1356 | 1.12 (0.97-1.29) |  | 0.70 (0.56-0.86) |  |
| ^a^ adjusted for all other variables in the table  ^b^ missing data excluded from test for trend in odds | | | | | |

| **Table 3: Adjusted and unadjusted Odds Ratios, including p values of predictors of D3 smokers in the Health Survey for England 2000-2010** | | | | | |
| --- | --- | --- | --- | --- | --- |
|  | Odds of fulfilling definition D3 criteria 2000-2010 | | | | |
|  | n | Unadjusted OR  (95% CI) | p-value for trend | Adjusted OR^a^  (95% CI) | p-value for trend |
| **Gender** |  |  |  |  |  |
| Female | 11134 | 1.00 | 0.004 | 1.00 | <0.001 |
| Male | 9748 | 1.12 (1.04-1.21) |  | 1.19 (1.10-1.28) |  |
| **Age group** |  |  |  |  |  |
| 26-34 | 4725 | 1.00 | <0.001 | 1.00 | <0.001 |
| 35-49 | 7940 | 1.47 (1.31-1.65) |  | 1.48 (1.32-1.67) |  |
| 50-59 | 3989 | 2.08 (1.84-2.36) |  | 2.08 (1.83-2.36) |  |
| 60+ | 4228 | 2.76 (2.44-3.11) |  | 2.69 (2.38-3.04) |  |
| **Socioeconomic status** |  |  |  |  |  |
| High | 5113 | 1.00 | <0.001 | 1.00 | <0.001 |
| Intermediate | 8261 | 1.34 (1.21-1.49) |  | 1.27 (1.15-1.42) |  |
| Low | 5582 | 1.81 (1.62-2.01) |  | 1.71 (1.53-1.91) |  |
| Missing^b^ | 1926 | 1.64 (1.41-1.89) |  | 2.19 (1.76-2.73) |  |
|  | | | | | |
| 2000 | 1728 | 1.00 | <0.001 | 1.00 | 0.040 |
| 2001 | 3235 | 1.09 (0.92-1.30) |  | 1.08 (0.91-1.29) |  |
| 2002 | 1562 | 1.22 (1.00-1.48) |  | 1.23 (1.01-1.49) |  |
| 2003 | 2992 | 1.13 (0.95-1.34) |  | 1.12 (0.94-1.33) |  |
| 2004 | 1288 | 1.18 (0.96-1.44) |  | 1.16 (0.94-1.43) |  |
| 2005 | 1519 | 1.11 (0.91-1.36) |  | 1.11 (0.91-1.36) |  |
| 2006 | 2576 | 1.23 (1.03-1.46) |  | 1.18 (0.99-1.40) |  |
| 2007 | 1241 | 1.17 (0.95-1.44) |  | 1.11 (0.90-1.37) |  |
| 2008 | 2598 | 1.37 (1.15-1.63) |  | 1.33 (1.12-1.58) |  |
| 2009 | 809 | 1.42 (1.13-1.79) |  | 1.35 (1.08-1.70) |  |
| 2010 | 1334 | 1.24 (1.01-1.51) |  | 0.73 (0.55-0.96) |  |
| ^a^ adjusted for all other variables in the table  ^b^ missing data excluded from test for trend in odds | | | | | |
